# Perioperative Management of a Case of Trousseau Syndrome Accompanied by Aortic Valve Vegetation

**DOI:** 10.7759/cureus.77202

**Published:** 2025-01-09

**Authors:** Mae Harada, Yuki Ochiai, Shigehito Sawamura

**Affiliations:** 1 Department of Anesthesia and Critical Care, Teikyo University School of Medicine, Tokyo, JPN

**Keywords:** direct oral anticoagulant, low-molecular-weight heparin, nonbacterial thrombotic endocarditis, perioperative management, trousseau syndrome

## Abstract

There is no established consensus on the appropriate perioperative management of patients with Trousseau syndrome. In particular, the selection of anticoagulants, including direct oral anticoagulants (DOACs), remains a critical point. Current guidelines recommend continuous administration of unfractionated or low-molecular-weight heparin but do not specify the efficacy of DOACs. We report a case of Trousseau syndrome and nonbacterial thrombotic endocarditis (NBTE) with recurrent embolism, focusing on perioperative management and anticoagulant selection.

In the case we encountered, a previously healthy woman presented with recurrent cerebral infarctions and was incidentally found to have an aortic valve mass, suggestive of papillary fibroelastoma. However, the planned resection of the aortic valve mass was canceled because transesophageal echocardiography (TEE) after the induction of general anesthesia confirmed its disappearance. The patient was discharged without thrombotic events but was readmitted three weeks later due to recurrent cerebral infarction. The embolisms recurred despite treatment with Edoxaban but improved with continuous heparin infusion, suggesting that DOACs were ineffective in this case. Further investigation revealed ovarian cancer, confirming Trousseau syndrome and NBTE as the underlying causes. Oophorectomy was performed, and no new ischemic events have occurred since. TEE evaluations were conducted at key decision-making points for surgery and other treatment strategies.

In conclusion, DOACs may be ineffective in some cases of Trousseau syndrome and NBTE. Continuous heparin infusion, along with frequent TEE monitoring of valvular vegetation, may help avoid unnecessary valvular surgery and enable prioritization of treatment for the underlying disease.

## Introduction

Trousseau syndrome refers to episodes of systemic embolism in patients with malignancy. Although the mechanism of thromboembolism is not fully understood, nonbacterial thrombotic endocarditis (NBTE) and resulting valvular vegetations are sometimes observed.

In this case, we managed the perioperative care of a patient with Trousseau syndrome who experienced recurrent embolic events caused by NBTE of the aortic valve. Despite these complications, the patient successfully underwent surgery for a gynecological malignancy. To the best of our knowledge, there is only one other report on the perioperative management of a patient with Trousseau syndrome who underwent gynecological surgery successfully one month after a massive cerebral infarction [1].

Our case, however, involved not only Trousseau syndrome but also recurrent NBTE-related embolic events during the perioperative period. This case provides important insights into anticoagulant therapy, the timing of surgery, and the use of transesophageal echocardiography (TEE). Regarding anticoagulant therapy, there is no consensus on the efficacy of direct oral anticoagulants (DOACs) in patients with Trousseau syndrome [2], although some reports question their effectiveness. In this case, DOAC therapy appeared to be ineffective [3,4].

This case report offers valuable insights into the perioperative management of Trousseau syndrome and NBTE, contributing to the growing body of evidence in this field.

## Case presentation

A previously healthy 66-year-old woman, 163 cm in height and weighing 47 kg, presented with dizziness and was diagnosed with a cerebral infarction. During the evaluation, an incidental splenic infarction was identified on thoracoabdominal computed tomography (CT). Edoxaban, a DOAC, was initiated at a dose of 30 mg/day.

**Figure 1:**
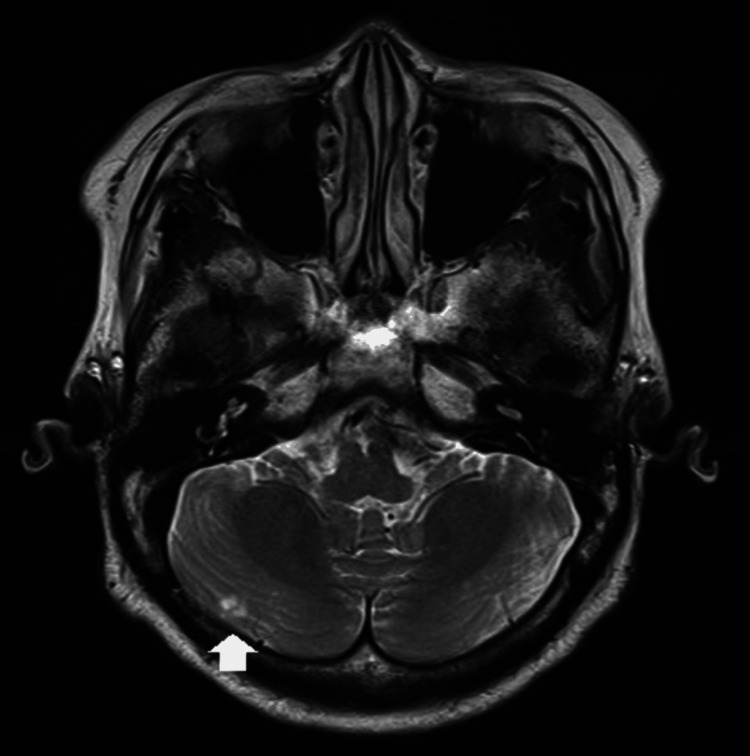
Head MRI T2-weighted image. A novel cerebral infarction was identified in the right cerebellum (arrow).

**Figure 2:**
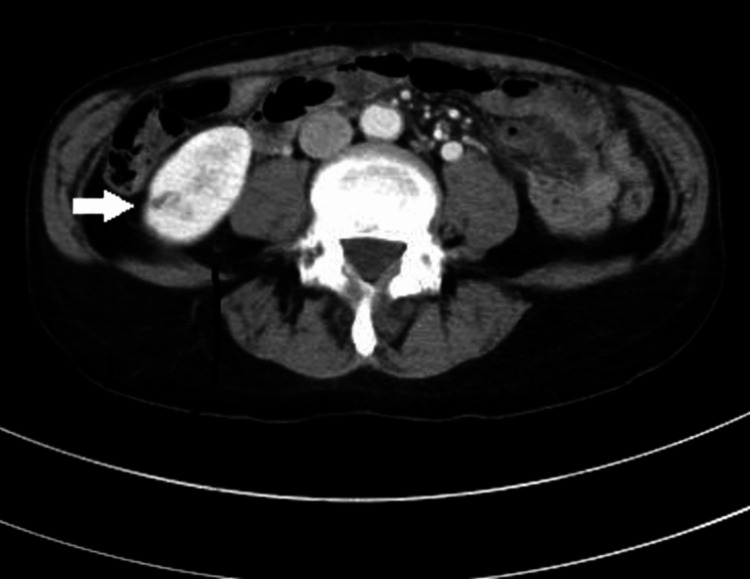
Contrast-enhanced abdominal CT image A small infarction was observed in the right kidney (arrow).

**Figure 3:**
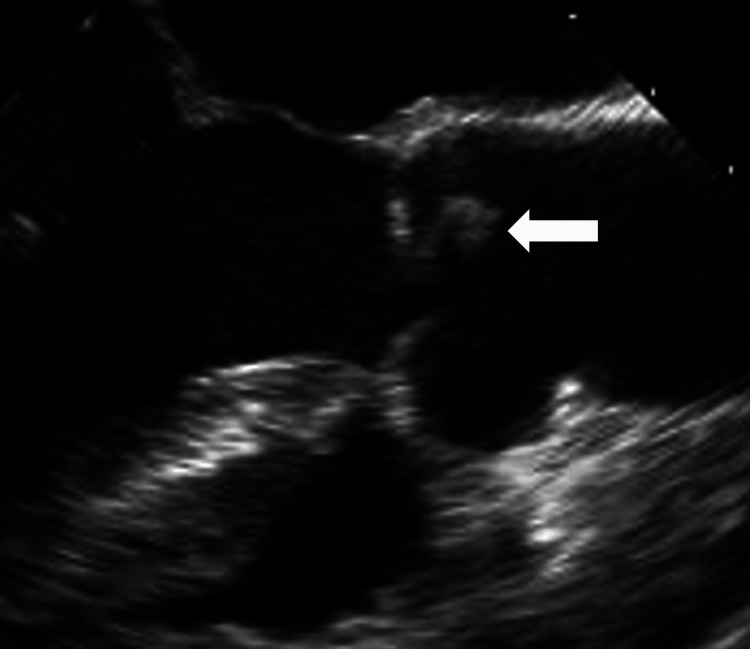
Transesophageal echocardiography image with the mid-esophageal aortic valve long axis view A pedunculated, oval-shaped, isoechoic, and mobile mass at the non-coronary cusp of the aortic valve was revealed (arrow).

Two months later, she developed new-onset disorientation and slurred speech. Imaging studies revealed a new cerebral infarction and a renal infarction, as shown in Figures 1, 2. She was admitted to the hospital, and Edoxaban was switched to continuous intravenous heparin at a dose of 10,000 units/day. During the evaluation of multiple infarctions, transthoracic echocardiography (TTE) and TEE revealed a pedunculated, oval-shaped, isoechoic mass measuring approximately 4×6 mm at the non-coronary cusp of the aortic valve, as shown in Figure 3. The mass had frayed edges and was mobile, suggesting a papillary fibroelastoma. Left ventricular function was normal, with no significant valvular disease or intracardiac thrombi observed. There were no findings suggestive of infective endocarditis, and there was no history of paroxysmal atrial fibrillation or similar conditions.

Further investigation revealed persistent thrombocytopenia and elevated levels of D-dimer and fibrin degradation products, as shown in Table 1, indicating findings consistent with chronic disseminated intravascular coagulation (DIC). After consultation with the hematology department and performing a bone marrow biopsy, no abnormalities were identified.

**Table 1:** Changes in platelet count, D-dimer, and FDP levels, and the occurrence of clinical events. The date of the first onset of cerebral infarction is defined as day 1. Persistent thrombocytopenia, along with elevated D-dimer and FDP levels, was observed, indicating findings consistent with chronic DIC. The elevation of D-dimer and FDP levels was correlated with embolic events. FDP: fibrin degradation product.

Day	Platelet count (×10^4^/μL) (reference range: 15.8-34.8)	D-dimer (μL/mL) (reference range: 0-0.9)	FDP (μL/mL) (reference range: 0-4.9)	Clinical events
1	17.7	36.2	Not available	First cerebral infarction
21	19.1	16.1	52.1	Identification of a splenic infarction
57	11.5	12.1	61	No special remarks
72	7.2	16.3	68	Second cerebral infarction and a renal infarction
81	28.7	2.4	3.6	Identification of an aortic valve mass
100	16.6	8.8	21.9	Aortic valve surgery→cancellation due to disappearance of an aortic valve mass
122	7.4	14	Not available	No special remarks
132	4.2	16.3	71.9	Third cerebral infarction
136	13.4	2.1	3.1	Emergence of a novel aortic valve vegetation
153	22.3	2.3	3.4	Oophorectomy
159	20.4	1.8	3.3	Postoperative day 6

Based on these findings, the papillary fibroelastoma was suspected to be the source of the emboli, and thoracoscopic aortic valve tumor resection under cardiopulmonary bypass was planned. After multidisciplinary discussions, it was decided to perform the surgery one month after the onset of the most recent cerebral infarction, considering the risk of perioperative intracranial hemorrhage. Additionally, due to the possibility of thrombus formation on the surface of the papillary fibroelastoma, continuous intravenous heparin was maintained until the surgery.

**Figure 4:**
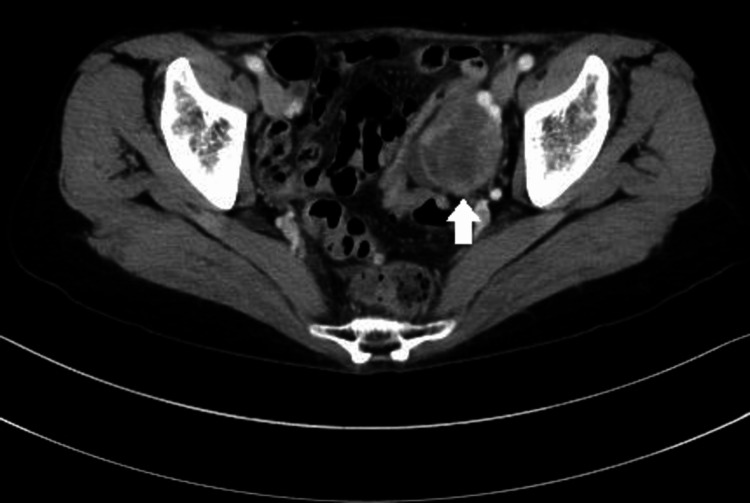
Contrast-enhanced pelvic CT image. A solid tumor, approximately 45 mm in size was observed in the left ovary (arrow).

As for other preoperative findings, contrast-enhanced CT of the pelvis revealed a solid tumor, approximately 45 mm in size, in the left ovary, as shown in Figure 4. A gynecological consultation was obtained, which showed no elevation in tumor markers, and the imaging results did not suggest malignancy.

After the induction of general anesthesia for aortic valve tumor resection, a TEE probe was inserted. TEE revealed that the aortic valve mass had disappeared, leaving only a slightly mobile, linear structure approximately 1 mm in size on the non-coronary cusp of the aortic valve. Following consultation, the surgery was canceled without any incisions being made. The patient awoke without complications, and no worsening of neurological symptoms was observed. Postoperative MRI of the head and thoracoabdominal CT showed no new infarctions.

At this point, two possible explanations for the disappearance of the mass were considered. One possibility was that the aortic valve tumor, such as a papillary fibroelastoma, had embolized and detached. The other was that the mass had been a thrombus that dissolved as a result of the preoperative continuous intravenous heparin infusion.

**Figure 5:**
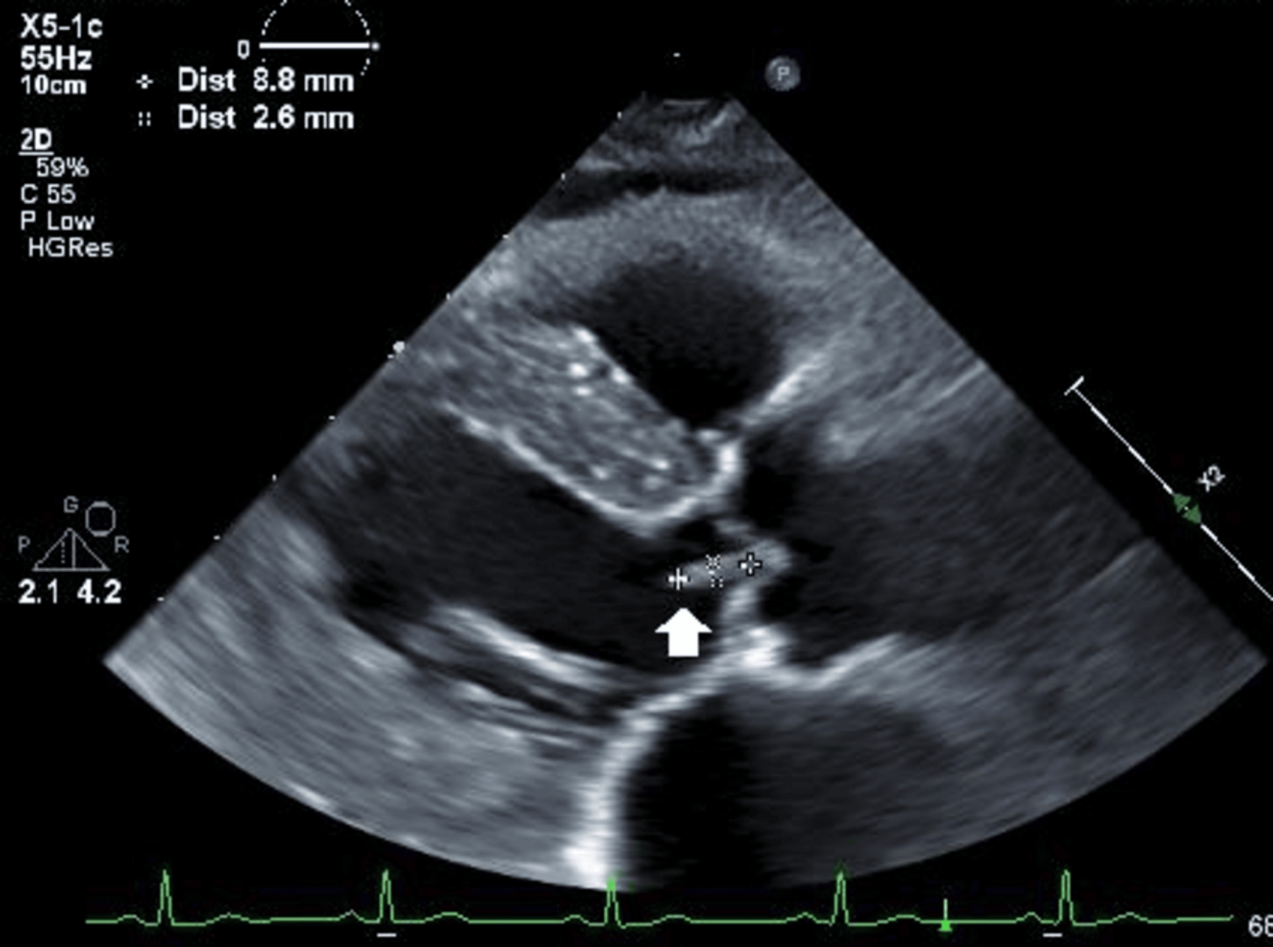
TTE image with parasternal long-axis view A new 9-mm mobile mass on the left coronary cusp of the aortic valve was revealed, suggesting a completely different lesion (arrow). TTE: transesophageal echocardiography.

Subsequently, continuous heparin administration was switched to Edoxaban at 30 mg/day, and the patient was discharged. However, three weeks later, the patient experienced a third episode of cerebral infarction. At this time, TTE and TEE revealed a new 9-mm mobile mass on the left coronary cusp of the aortic valve, as shown in Figure 5, suggesting a completely different lesion.

**Figure 6:**
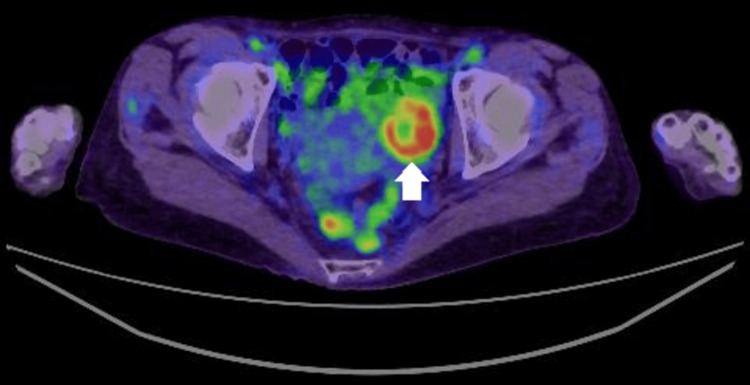
Pelvic positron emission-CT imaging Uptake in the solid component of the left ovarian tumor was observed (arrow).

During the detailed examination, it was observed that the ovarian tumor, previously considered benign, had shown a tendency to enlarge, with accumulation noted on positron emission tomography-CT (PET-CT), as shown in Figure 6. These findings raised suspicion of Trousseau’s syndrome associated with ovarian cancer. The recurrent aortic valve mass was considered to be NBTE associated with this condition. It was determined that performing an oophorectomy was essential for a definitive diagnosis. The decision was made to first manage the aortic valve NBTE with continuous heparin infusion at a dose of 10,000-15,000 units/day and proceed with oophorectomy after clinical improvement.

After 14 days of continuous heparin infusion, follow-up TEE revealed that the aortic valve NBTE had disappeared, and oophorectomy was planned under general anesthesia. The timing of the oophorectomy was set shortly after the disappearance of NBTE was confirmed by TEE, 15 days after the onset of the cerebral infarction. Following the induction of anesthesia, a TEE was performed, and no recurrence of NBTE was observed. The surgery was completed without complications. Continuous intravenous heparin infusion was resumed immediately after surgery, and three weeks postoperatively, it was switched to subcutaneous injections. Two months later, the patient was transitioned to warfarin, and no new ischemic events have occurred since. The pathological diagnosis was clear cell carcinoma, stage IA ovarian cancer.

Although a pathological diagnosis of the vegetations on the aortic valve could not be performed, it is reasonable to clinically conclude that the patient had Trousseau's syndrome with concurrent NBTE. This conclusion is supported by the patient's repeated embolic events, chronic DIC, the detection of vegetations on different heart valves via TEE on two occasions, and the complete resolution of embolic events following curative surgery for ovarian cancer.

**Figure 7:**
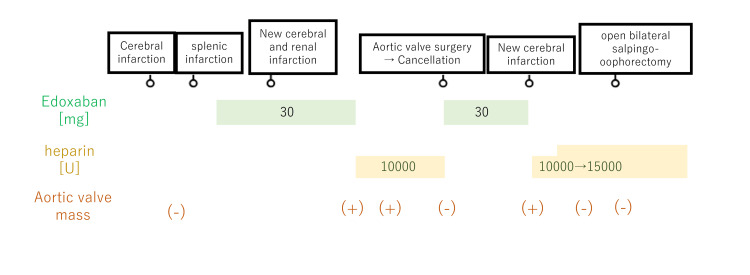
The clinical course of this case.

The clinical course of this case is shown in Figure 7.

## Discussion

Trousseau syndrome is a condition characterized by multiple thrombi throughout the body in cancer patients, sometimes accompanied by NBTE. NBTE is a pathological state in which fragile, sterile vegetations form on the heart valves, consisting of fibrin, platelets, and immune complexes. While embolism is a common complication, NBTE is frequently asymptomatic and is found in 0.9-1.6% of autopsies [5].

The exact mechanism of NBTE remains unclear; however, it is thought that increased levels of tumor necrosis factor and cytokines in patients with a hypercoagulable state cause endothelial cell injury, promoting vegetation formation. Unexplained arterial embolism, vegetations on the heart valves, and a tendency toward DIC in cancer patients are significant findings that raise suspicion for NBTE associated with Trousseau syndrome [6]. 

However, the diagnosis of this case was delayed due to the initial assumption that the ovarian tumor was benign and because the findings of the aortic valve tumor on TEE resembled those of a fibroelastoma.　

Characteristic clinical features of fibroelastoma include arising from the ventricular side of the valve or the endocardium of the atria or ventricles, typically occurring as solitary lesions with a diameter around 1 cm, being pedunculated with frequent mobility, and having a distinctive frond-like appearance [7].

In contrast, NBTE vegetations are less than 3 mm in diameter in 70% of cases and tend to be multiple. However, they can occasionally be larger than 10 mm. NBTE vegetations often have irregular borders, heterogeneous internal echogenicity, and may be accompanied by thickening of the valve cusps [8]. The mitral valve is the most common site, followed by the aortic valve. Due to the generally small size of these lesions, detection via TTE is often challenging [9]. When NBTE is suspected, TEE, which has superior sensitivity and specificity, is recommended.

In this case, even after the diagnosis of NBTE, TEE evaluations were conducted at key decision-making points for surgery and other treatment strategies, proving to be valuable. Specific points that were effective in this case include: (1) Performing TEE in patients with Trousseau syndrome who experience recurrent embolism. (2) If NBTE is detected by TEE, initiating continuous heparin infusion and re-evaluating with TEE after two weeks to confirm improvement and determine a new course of action. (3) Since NBTE can recur even after initial improvement, if surgery is planned, re-evaluating with TEE after the induction of general anesthesia is recommended to ensure no recurrence.

Numerous reports suggest that Trousseau syndrome and NBTE can improve with treatment of the underlying disease [6]. If surgery for the primary condition is feasible, it should be performed as early as possible. However, in cases with concurrent cerebral infarction, non-cardiac surgery performed within three months of the infarction carries a high risk of hemorrhagic stroke. In previous case reports of gynecologic malignancies with cerebral infarction caused by Trousseau syndrome [1], the timing of surgery was determined by carefully balancing the risk of vascular complications against the benefits of early surgical intervention. In one such case, surgery for the gynecologic malignancy was performed one month after the cerebral infarction without complications. In the present case, the patient experienced recurrent embolic events caused by NBTE and had a relatively mild cerebral infarction, suggesting significant benefits of early surgery. Consequently, surgery was planned as soon as NBTE showed improvement. Surgery for the gynecologic malignancy was performed on the 15th day after the cerebral infarction without complications. While early surgery is desirable in such cases, its timing should be carefully determined based on the severity of the cerebral infarction and other relevant factors.

Regarding valve surgery in cases of NBTE, NBTE lesions are typically small and rarely impair valve function. As a result, valve surgery is seldom required [10]. However, NBTE carries a high risk of embolism, and if embolism cannot be controlled with adequate antithrombotic therapy, valve surgery should be considered a priority, akin to the approach for infective endocarditis. Unlike infective endocarditis, where the removal of infected tissue is mandatory, valve preservation may be possible in NBTE.

For antithrombotic therapy in NBTE, current recommendations from the American College of Chest Physicians advocate for the use of unfractionated or low-molecular-weight heparin [2]. These recommendations are based on evidence comparing these therapies to warfarin. However, the guidelines do not address the efficacy of DOACs.

A recent systematic review on the prevention of recurrent venous thromboembolism (VTE) in cancer patients has shown that DOACs are more effective than low-molecular-weight heparin in preventing VTE recurrence [11]. However, no definitive recommendations exist regarding their efficacy in Trousseau syndrome or NBTE. Recent case reports have indicated that embolic events due to NBTE could not be prevented under DOAC therapy but improved after switching to heparin [3,4], consistent with the findings in the present case.

The mechanism of hypercoagulability in Trousseau syndrome is complex, but heparin may exert beneficial effects on NBTE associated with Trousseau syndrome due to its multifaceted action, including inhibition of coagulation via antithrombin and factor Xa [12]. Further studies involving more cases are needed to establish appropriate antithrombotic strategies for NBTE.

## Conclusions

In preoperative evaluations of cancer patients, if unexplained arterial embolism or valvular vegetations are observed, embolic events due to NBTE should be suspected. In such cases, consideration should be given to the possibility that DOACs may not be effective. Adequate continuous intravenous administration of heparin, along with frequent TEE to monitor for tumor reduction or resolution, may allow for the avoidance of unnecessary valve surgery and prioritize the treatment of the underlying disease.
